# COVID-19 Related Experience, Knowledge, Attitude, and Behaviors Among 2,669 Orthodontists, Orthodontic Residents, and Nurses in China: A Cross-Sectional Survey

**DOI:** 10.3389/fmed.2020.00481

**Published:** 2020-08-07

**Authors:** Fang Hua, Danchen Qin, Jiarong Yan, Tingting Zhao, Hong He

**Affiliations:** ^1^Department of Orthodontics, Hubei-MOST KLOS & KLOBM, School & Hospital of Stomatology, Wuhan University, Wuhan, China; ^2^Center for Evidence-Based Stomatology, Hubei-MOST KLOS & KLOBM, School & Hospital of Stomatology, Wuhan University, Wuhan, China; ^3^Division of Dentistry, Faculty of Biology, Medicine and Health, Manchester Academic Health Science Centre, School of Medical Sciences, University of Manchester, Manchester, United Kingdom

**Keywords:** COVID-19, coronavirus, infection control, orthodontics, questionnaire

## Abstract

**Objectives:** To assess the current COVID-19 related experiences, knowledge, attitudes, and behaviors among orthodontists, orthodontic residents, and orthodontic nurses in China, and to identify factors associated with their self-perceived and actual level of knowledge, as well as their willingness to treat/care for COVID-19 patients.

**Materials and Methods:** A cross-sectional online survey was conducted in China using a 37-item questionnaire developed based on previous research. A professional online survey tool (www.wjx.cn) and a social media platform (WeChat) were used to display and distribute the questionnaire. Data were collected during April 11 to 13, 2020, when most regions of China had resumed dental practice except for high-risk regions such as Wuhan. Then the data were analyzed with multivariable generalized estimating equations.

**Results:** A total of 2,669 valid questionnaires were collected. Orthodontic services were suspended for nearly all respondents (97.8%) during the epidemic, and 68.0% had resumed work by the time they completed the questionnaire. The majority of respondents (80.2%) were confident that they understood COVID-19 related knowledge, but most of them only correctly answered less than half of the questions testing their actual level of knowledge. About two-thirds (64.1%) were willing to treat/care for patients with confirmed or suspected COVID-19. The completion of relevant training programs was significantly associated with more confidence in knowledge mastery (*P* < 0.001) and a higher actual level of knowledge (*P* < 0.001), but did not increase their willingness to treat/care for patients with COVID-19 (*P* = 0.235).

**Conclusions:** Before work resumption, COVID-19-related training programs are essential for the improvement of knowledge, confidence, and preparedness of orthodontic professionals. Sufficient and proper protection should also be provided to ensure safety and reduce the psychological burden on them.

**Clinical Relevance:** The findings can provide evidence for policy-making related to the resumption of elective dental services.

## Introduction

After being discovered in Wuhan, China, last December, the coronavirus disease 2019 (COVID-19), caused by severe acute respiratory syndrome coronavirus 2 (SARS-CoV-2), has spread quickly to most parts of the world (1). The World Health Organization (WHO) officially declared the COVID-19 outbreak a public health emergency of international concern on January 30, 2020 (2), and then a global pandemic on March 11, 2020 (3). As of May 29, 2020, there have been a total of 5,701,337 laboratory-confirmed COVID-19 cases and 357,688 deaths in 217 countries, territories, or areas (4).

Current observations have suggested that people of all ages are generally susceptible to COVID-19. However, those who are in close contact with confirmed cases or asymptomatic carriers, including health care workers (HCWs) and other patients within hospitals and/or clinics, are at higher risk of infection (5). As of February 24, 2020, a total of 3,387 HCWs from 476 medical institutions across China were reported to be infected with SARS-CoV-2 (including 2,055 confirmed cases, 1,070 clinically diagnosed cases, and 157 suspected cases), among whom over 90% were from Hubei province and over 20 had died (6, 7). More recently, the WHO announced that there had been at least 22,073 COVID-19 cases among HCWs from 52 countries by April 8, 2020 (8). However, currently there are no data available for cross-transmission in dental settings or cases reported among dental HCWs (9, 10).

As many dental procedures can generate a large number of droplets and aerosols, standard protective measures used in daily practice are not enough to prevent cross-infection among dental practitioners and dental patients (11). In light of this, most provinces and cities in China released regulations in late January, which explicitly restricted dental services to emergency care only, and suspended all elective treatments until further notice (11). As a result of these regulations, as well as public health interventions such as traffic suspension and home quarantine (12), many dental professionals stayed at home during the epidemic, communicating with existing and potential new patients via smartphones and online consultation platforms (13, 14).

Combined, the above-mentioned changes in work and life due to COVID-19 can have a huge impact on HCWs, including non-frontline workers. Findings of a recent study have suggested that the level of psychological stress among non-frontline nurses could be significantly higher than frontline nurses (15). In addition, as China moves from the 2-month containment phase to the mitigation stage (16), the preparedness of non-frontline HCWs to resume work is of great importance. Two recent studies have investigated the knowledge and attitudes regarding COVID-19 among Chinese HCWs in psychiatric hospitals (17) and Jordanian dentists (18), respectively. However, to our knowledge, similar research has not been carried out among HCWs in the field of orthodontics.

Therefore, the objectives of this study were: (1) to assess the current COVID-19-related experiences, knowledge, attitudes, and behaviors among orthodontists, orthodontic residents, and orthodontic nurses in China; (2) to identify factors associated with the self-perceived and actual level of knowledge; and (3) to explore the association between willingness to treat/care for patients with confirmed or suspected COVID-19 and potentially related factors.

## Materials and Methods

### Ethical Approval

This study was a convenience-sample open online survey, written in accordance with the CHEcklist for Reporting Results of Internet E-Surveys (CHERRIES) guidelines (19). The study protocol was approved by the Ethics Committee of School & Hospital of Stomatology, Wuhan University (No. 2020-B25). Informed consent was obtained from all respondents. A brief introduction to the study was provided in the first part of the questionnaire, including the target population, study objectives, the time needed to complete the questionnaire, as well as the names and contact information of the investigators. Participation in the survey was voluntary, with neither reward nor penalty. All respondents were informed that they were free to continue or quit at any time, and the submission of the questionnaire would be regarded as consent to participate. To protect respondents' privacy, the survey was anonymous, and all the raw data were stored in one author's computer and kept confidential.

### Survey Design

A 37-item questionnaire (Supplementary Material 1) was developed according to previous research on knowledge, attitudes, and behaviors of HCWs (17, 20). We added several questions for background information of the participants, and rephrased questions to make them relevant to orthodontics; the construction of the measured concepts and scoring methods remained the same. The survey consisted of four aspects: background information, knowledge, attitudes, and behaviors. The first 16 questions were regarding the background characteristics, including seven for demographics, five for workplace information, and four for COVID-related personal experience. Ten questions were in the knowledge section, including three Likert-scale items (#17, #19, and #20) for the self-perceived level of knowledge, one semi-open question (#18) for their source of knowledge, and six (3 single-choice and 3 multiple-choice, #21 to #26) questions for the assessment of actual knowledge level. Among five questions in the attitude section, three were Likert-scale (#27 to #29) to evaluate the attitudes toward personal protective equipment (PPE) in the context of COVID-19. In addition, respondents were asked a yes-no question (#30) about their willingness to treat/care for orthodontic patients with confirmed or suspected COVID-19, and if “no” was selected, they should explain the reasons for this choice (a semi-open question, #31). The behavior section contained six questions. Respondents were required to rate their actual or expected behaviors after the resumption of orthodontic services for five Likert-scale questions (#32, #33, and #35 to #37), and their compliance to PPE on a percentage scale (#34). For all Likert-scale questions, a five-point Likert scale was used ranging from 1 (completely disagree) to 5 (completely agree). In addition, each aspect contained several Likert-scale items to ensure reliability and validity.

### Participant Recruitment

Eligible participants were orthodontists, orthodontic residents, orthodontic nurses, and general dental practitioners and nurses offering orthodontic care. The sample size was calculated using an online calculator (www.raosoft.com/samplesize.html). With a 5% margin of error and a 95% confidence level, the required sample size was 377.

Prior to its official release, the questionnaire was sent to a small group of around 20 orthodontic professionals to record the time needed to complete it and check if the questions were clear and unambiguous. After this pilot survey, the questionnaire was distributed and publicized to orthodontic professionals nationwide.

We used purposive and snowball sampling routes to recruit participants. The survey was distributed from April 11 to 13, 2020, when most regions of China had resumed dental practice, except for high-risk regions such as Wuhan. We first disseminated the questionnaire link through orthodontics-related blogs on WeChat (Tencent, Shenzhen, China), which has been the most widely used social media platform in China (21). Then we sent messages to chat groups of orthodontic practitioners and asked them to invite their friends, engaging orthodontics to participate. Also, professor H.H invited her professional network personally.

The questionnaire was collected using a professional online survey platform (www.wjx.cn). Since respondents could submit their answers only when all the questions are finished, this platform guaranteed the completeness of the collected questionnaires. To prevent multiple answers from the same individual, the questionnaire could only be accessed through WeChat, and each WeChat account could only answer once. The respondents could not change their answers once submitted. All valid responses were included, and the expected valid response rate was 90% after excluding invalid answers. As planned *a priori*, questionnaires with incomplete responses, apparent errors, and atypical timestamps (<2 min or more than 30 min) (22) were considered invalid and excluded.

### Statistical Analysis

The original data were downloaded from the online survey platform. For the semi-open question, reasons for their unwillingness to treat/care for COVID-19 patients (#31), themes were manually extracted and coded by three authors (F.H., D.Q., and T.Z.), independently and in duplicate, with all discrepancies resolved by discussion. Each response could be coded into multiple themes if containing miscellaneous information, and then the count for each theme were calculated. The self-perceived level of knowledge about COVID-19 was evaluated by averaging the scores of three Likert-scale items (#17, #19, and #20). Regarding the actual level of knowledge, we gave 1 point for each question of #21 to #26 answered correctly, and no point for any item incorrectly answered. Then a total knowledge score (score range: 0 to 6) for each respondent was calculated by totaling the scores of all these six items.

Descriptive statistics were performed to summarize the characteristics and the answers of each question. Categorical data were presented as counts and percentages. Continuous data were expressed as means ± standard deviations (SDs) and range, and skewed data and Likert-scale responses were presented as medians and interquartile ranges. Chi-square tests and Fisher's exact tests were used to analyze categorical data, with pairwise comparisons adjusted by Bonferroni correction. Likert-scale questions were analyzed with Kruskal-Wallis tests, followed by Dunn's tests for *post-hoc* pairwise comparisons. The self-perceived level of knowledge and the total knowledge score were compared using one-way analysis of variance (ANOVA) with Tukey adjustment.

Generalized estimating equations (GEE) regression analyses were performed to explore factors associated with the self-perceived level of knowledge, total knowledge score, and the willingness to treat/care for COVID-19 patients (#30). Robust estimate of covariance and the independent correlation matrix were adopted. For continuous outcomes (the self-perceived level of knowledge and the total knowledge score), we used a GEE with a normal distribution for the response variables and identity link; for the binary outcome (the willingness to treat/care for COVID-19 patients), we used a GEE with a binomial distribution and logit link. As determined *a priori*, all the characteristics in the first part of the questionnaire (#1 to #16) were served as independent variables, namely age, gender, profession, years of practice, academic degree, marital status, cohabitants, location of workplace, settings, provision of online orthodontic consultation, current status of orthodontic services at the workplace, participation in anti-epidemic activities, current status of personal orthodontic practice, completion of any COVID-19-related training program, and experience of treating/caring for COVID-19 patients. In addition, self-perceived level of knowledge and total knowledge score also served as the predictors of the willingness to treat/care for COVID-19 patients. In these analyses, we carried out univariable analyses first, and then entered all significant factors into multivariable analyses. Tolerance and variance inflation factor (VIF) were used to detect multicollinearity, and predictors with tolerance <0.1 or VIF > 10 were excluded from the final model. Two-sided *P* < 0.05 was considered statistically significant.

## Results

Due to the character of our recruitment methods, it was impossible to estimate the total number of orthodontic practitioners who had received our survey invitation. Among the 2,890 questionnaires collected, 221 responses were invalid according to the predetermined eligibility criteria: four failed to complete the survey before the deadline, 15 submitted with an unusual completion time, and 202 questionnaires had obvious mistakes such as abnormal age and contradictory answers to similar questions. After removing these invalid responses, 2,669 questionnaires from 2,669 respondents were included in analyses, resulting in a valid response rate of 92.4%.

### General Information

**Figure 1:**
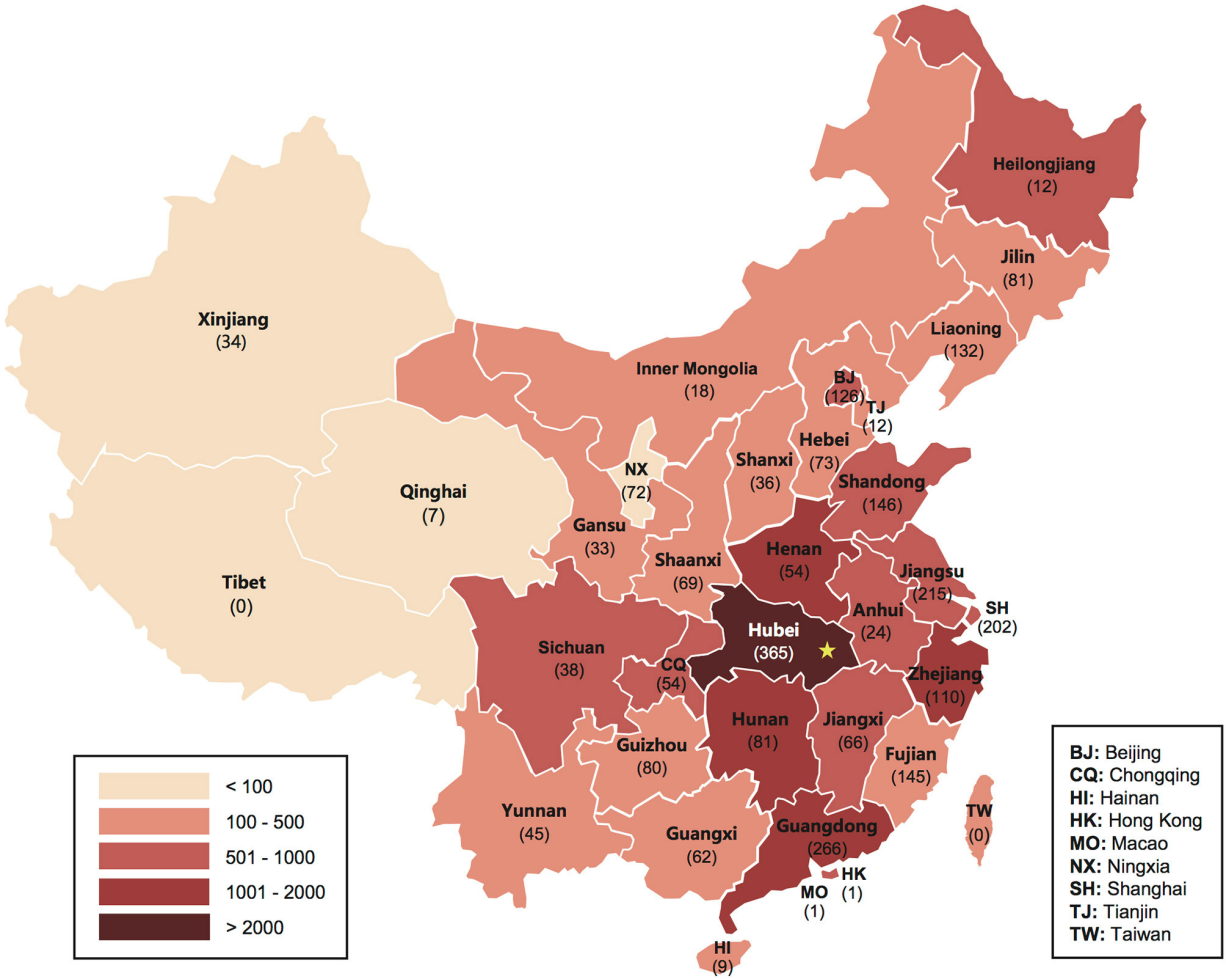
The geographical distribution of respondents to this survey (*n* = 2,669). Gradient of colors represents the cumulative number of confirmed COVID-19 cases in each provincial-level administrative region (as of April 10, 2020). Numbers in parentheses indicate the number of respondents from each region. The star marks the location of Wuhan.

The respondents came from 32 of the 34 provincial-level administrative regions of China. Figure 1 illustrates the geographical distribution of respondents together with the cumulative number of confirmed COVID-19 cases in each region as of April 10. There were 739 (27.7%) males and 1,930 (72.3%) females between 19 and 62, with an average age of 34.3 (SD, 8.5). The majority of the respondents were orthodontists (64.8%), followed by orthodontic residents/postgraduate students (20.5%) and orthodontic nurses (14.6%). Nearly half (47.7%) of them had a relatively short working experience in orthodontics (≤5 years). The majority (91.0%) lived with other people, and nearly three-fifths (58.2%) lived with their parents and/or children.

As shown in Table 1, almost all respondents (97.8%) stated that the dental hospitals or clinics in which they worked had full (49.2%) or partial (48.6%) suspension of orthodontic treatment during the epidemic. Also, a majority of them (87.4%) stated that their workplaces provided online orthodontic consultation to either existing orthodontic patients only (30.9%), or both existing and potential new orthodontic patients (56.5%). In addition, 87.1% of the respondents reported that their workplaces had recently resumed orthodontic services in full (43.0%) or in part (44.1%).

**Table 1:** Demographic and workplace information of the respondents to this survey.

**Characteristics**	***N***	**%**
**Demographics**		
Age^§^	34.3 ± 8.5	19–62
Gender		
Female	1,930	72.3
Male	739	27.7
Profession		
Orthodontist	1,731	64.8
Orthodontic resident/postgraduate student	547	20.5
Orthodontic nurse	391	14.6
Years of orthodontic practice		
≤5 years	1,274	47.7
5–10 years	620	23.2
10–20 years	516	19.3
>20 years	259	9.7
Highest degree		
PhD	211	7.9
Master	778	29.1
Bachelor	1,371	51.4
Junior college	297	11.1
Technical secondary school	12	0.4
Marital status		
Single	944	35.4
Married, without children	240	9.0
Married, with children	1,465	54.9
Others	20	0.7
Cohabitants		
Live alone	241	9.0
Parents and/or children	1,553	58.2
Others	875	32.8
**Workplace information**		
Location		
Wuhan	267	10.0
Hubei province (excluding Wuhan)	98	3.7
Others	2,304	86.3
Setting		
Public	1,980	74.2
Private	689	25.8
Orthodontic services during epidemic		
Complete suspension	1,313	49.2
Partial suspension	1,297	48.6
No suspension	59	2.2
Online consultation		
No	337	12.6
Provided, only to existing orthodontic patients	824	30.9
Provided, to both existing and potential new orthodontic patients	1,508	56.5
Current status of orthodontic services		
Not resumed yet	338	12.7
Partially resumed	1,176	44.1
Completely resumed	1147	43.0
No suspension	8	0.3
**Total**	2,669	100.0

§ *Displayed as mean ± SD, and range*.

### Personal Experience

Table 2 shows the respondents' COVID-19-related personal experience during the epidemic. Although 28.6% of them had participated in anti-epidemic activities, including supporting fever clinics or designated COVID-19 hospitals, and acting as community volunteers, only 3.9% had the experience of treating or caring for patients with confirmed COVID-19. Approximately 80% of the orthodontists and nurses had resumed their orthodontic practice, whereas only one-fifth (19.4%) of the orthodontic residents had returned to work. Additionally, the majority of orthodontists (84.4%) and nurses (90.3%) had finished COVID-19-related training programs, while only 55.2% of orthodontic residents received relevant training.

**Table 2:** Respondents' personal experience during the COVID-19 epidemic by profession.

**Personal experience^§^**	**Total (*n =* 2,669)**	**Orthodontists (*n =* 1,731)**	**Orthodontic residents (*n =* 547)**	**Orthodontic nurses (*n =* 391)**	***P-*value^†^**
Participation in anti-epidemic activities^¶^					**<0.001**
No	1,907 (71.4)	1,150 (66.4)^a^	490 (89.6)^b^	267 (68.3)^a^	
Yes	762 (28.6)	581 (33.6)^a^	57 (10.4)^b^	124 (31.7)^a^	
Current status of orthodontic practice					**<0.001**^‡^
Not resumed yet	849 (31.8)	326 (18.8)^a^	441 (80.6)^b^	82 (21.0)^a^	
Resumed, less than 2 weeks	478 (17.9)	379 (21.9)^a^	46 (8.4)^b^	53 (13.6)^c^	
Resumed, 2 to 4 weeks	725 (27.2)	586 (33.9)^a^	38 (6.9)^b^	101 (25.8)^c^	
Resumed, more than 4 weeks	611 (22.9)	436 (25.2)^a^	22 (4.0)^b^	153 (39.1)^c^	
No suspension	6 (0.2)	4 (0.2)^a^	0 (0.0)^a^	2 (0.5)^a^	
Completion of COVID-19 related training program					**<0.001**
No	553 (20.7)	270 (15.6)^a^	245 (44.8)^b^	38 (9.7)^c^	
Yes	2,116 (79.3)	1,461 (84.4)^a^	302 (55.2)^b^	353 (90.3)^c^	
Experience of treating or caring for COVID-19 patients					**<0.001**^‡^
No	2,564 (96.1)	1,652 (95.4)^a^	543 (99.3)^b^	369 (94.4)^a^	
Yes	105 (3.9)	79 (4.6)^a^	4 (0.7)^b^	22 (5.6)^a^	

§ *Displayed as N (%)*.

¶ *Including activities in fever clinics or designated hospitals for COVID-19 patients, as community volunteers, and other COVID-19-related works*.

† *P-values in bold are statistically significant (<0.05)*.

‡ *Fisher' exact test*.

a, b, c *Groups with the same letters in the same row are not statistically different (P > 0.05) according to post hoc tests*.

As listed in Supplementary Table 1, the proportion of respondents that had not resumed orthodontic work was significantly different among location groups (*P* < 0.001). The highest proportion occurred in Wuhan (93.3%), followed by other parts of Hubei province (52.0%) and regions outside Hubei (23.8%). Accordingly, the proportion of respondents who had not completed COVID-19-related training programs was significantly higher in Wuhan (29.2%) than places outside Hubei (19.8%). In addition, the experience of treating or caring for COVID-19 patients was more common among respondents from Wuhan (9.0%) and other parts of Hubei (9.2%) than those from areas outside Hubei province (3.1%).

### Knowledge

**Figure 2:**
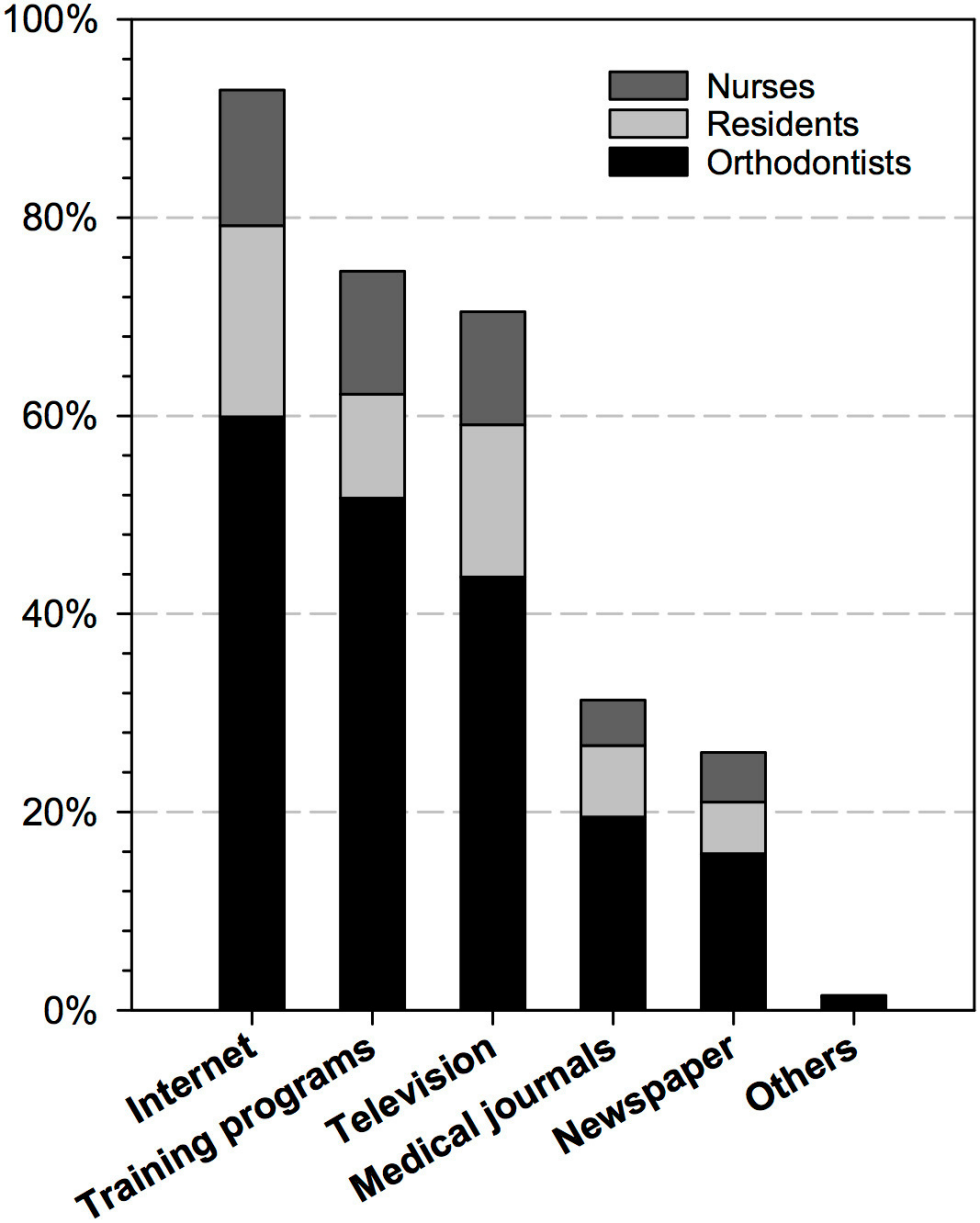
The sources of COVID-19-related knowledge among respondents (semi-open question, #18) (*n* = 2,669).

As described in Supplementary Table 2, the mean self-perceived level of knowledge of COVID-19 was 4.03 (SD 0.65), which means the respondents agreed that they were confident in their knowledge. Most respondents (80.2%) expressed agreement or complete agreement that they understood COVID-19-related knowledge. In addition, most were confident that they understood the risk of COVID-19 to patients and HCWs (84.1%), as well as how to protect themselves and patients during the epidemic (76.1%). As shown in Figure 2, the Internet (92.8%) was the respondents' primary source of knowledge, followed by training programs (74.6%), television (70.5%), medical journals (31.3%), and newspapers (26.0%). However, the mean total knowledge score was 2.74 (SD, 0.85), which suggests the respondents only correctly answered less than half of the six questions testing their actual level of knowledge on average.

Table 3 shows the results of GEE regression analyses for the self-perceived level of knowledge about COVID-19. According to multivariable analysis, respondents who had finished COVID-19-related training programs had significantly more confidence in knowledge mastery than those who had not (*B* = 0.406, 95% CI: 0.341 to 0.471, *P* < 0.001). In addition, a significantly higher self-perceived level of knowledge was also found in orthodontic nurses (*P* < 0.001), older people (*P* = 0.008), male individuals (*P* < 0.001), those who were living with parents and/or children (*P* = 0.024), those who suspended their orthodontic practice during the epidemic (*P* = 0.029), and those who had the experience of treating or caring for COVID-19 patients (*P* = 0.040).

**Table 3:** Results of univariable and multivariable generalized estimating equations (GEE) regression analyses for self-perceived level of knowledge.

**Variable**	**Univariable analysis**			**Multivariable analysis**		
	***B***	**95% CI**	***P*-value^¶^**	***B***	**95% CI**	***P-*value^¶^**
**Demographics**						
Age	0.012	(0.009, 0.015)	**<0.001**	0.008	(0.002, 0.014)	**0.008**
Gender						
Female	Reference			Reference		
Male	0.112	(0.058, 0.166)	**<0.001**	0.107	(0.054, 0.160)	**<0.001**
Profession			**<0.001**			**<0.001**
Orthodontist	Reference			Reference		
Orthodontic resident/postgraduate student	−0.264	(−0.325, −0.203)	**<0.001**	−0.012	(−0.100, 0.077)	0.797
Orthodontic nurse	0.094	(0.023, 0.164)	**0.009**	0.148	(0.070, 0.225)	**<0.001**
Years of orthodontic practice			**<0.001**			0.370
≤5 years	Reference			Reference		
5–10 years	0.095	(0.033, 0.158)	**0.003**	−0.059	(−0.128, 0.011)	0.099
10–20 years	0.184	(0.119, 0.249)	**<0.001**	−0.051	(−0.137, 0.035)	0.246
>20 years	0.259	(0.178, 0.339)	**<0.001**	−0.030	(−0.162, 0.102)	0.656
Academic degree			0.430			
Marital status			**<0.001**			0.090
Single	Reference			Reference		
Married, without children	0.170	(0.085, 0.255)	**<0.001**	0.078	(−0.011, 0.167)	0.088
Married, with children	0.191	(0.138, 0.245)	**<0.001**	−0.046	(−0.133, 0.042)	0.308
Others	0.095	(−0.247, 0.437)	0.586	0.005	(−0.344, 0.355)	0.976
Cohabitants			**<0.001**			**0.030**
Live alone	Reference			Reference		
Parents and/or children	0.141	(0.045, 0.234)	**0.004**	0.113	(0.015, 0.212)	**0.024**
Others	0.007	(−0.092, 0.106)	0.895	0.048	(−0.047, 0.144)	0.323
**Workplace information**						
Location			**0.020**			0.191
Wuhan	Reference			Reference		
Hubei province (excluding Wuhan)	0.198	(0.059, 0.338)	**0.005**	0.121	(−0.010, 0.252)	0.069
Others	0.078	(−0.008, 0.164)	0.075	0.056	(−0.052, 0.165)	0.309
Setting			0.612			
Orthodontic services during epidemic			**0.003**			0.130
Complete suspension	Reference			Reference		
Partial suspension	−0.040	(−0.090, 0.010)	0.313	−0.040	(−0.089, 0.010)	0.116
No suspension	0.225	(0.063, 0.387)	**0.032**	0.084	(−0.089, 0.257)	0.342
Online consultation			0.158			
Current status of orthodontic services			**<0.001**			**<0.001**
Not resumed yet	Reference			Reference		
Partially resumed	−0.018	(−0.098, 0.062)	0.658	−0.073	(−0.182, 0.037)	0.193
Completely resumed	0.081	(0.001, 0.161)	**0.047**	−0.045	(−0.163, 0.073)	0.452
No suspension	0.711	(0.388, 1.034)	**<0.001**	0.672	(0.463, 0.881)	**<0.001**
**Personal experience**						
Participation in anti-epidemic activities						
No	Reference			Reference		
Yes	0.160	(0.107, 0.213)	**<0.001**	−0.073	(−0.182, 0.037)	0.193
Current status of orthodontic practice			**<0.001**			**0.048**
Not resumed yet	Reference			Reference		
Resumed, less than 2 weeks	0.167	(0.096, 0.238)	**<0.001**	0.054	(−0.036, 0.145)	0.241
Resumed, 2 to 4 weeks	0.206	(0.143, 0.270)	**<0.001**	0.056	(−0.033, 0.145)	0.217
Resumed, more than 4 weeks	0.241	(0.173, 0.309)	**<0.001**	0.073	(−0.024, 0.169)	0.140
No suspension	0.728	(0.335, 1.120)	**<0.001**	−0.402	(−0.762, −0.042)	**0.029**
Completion of COVID-19 related training program						
No	Reference			Reference		
Yes	0.478	(0.417, 0.539)	**<0.001**	0.406	(0.341, 0.471)	**<0.001**
Experience of treating or caring for COVID-19 patients						
No	Reference			Reference		
Yes	0.277	(0.154, 0.399)	**<0.001**	0.137	(0.006, 0.268)	**0.040**

¶ *P-values in bold are statistically significant (<0.05)*.

However, in terms of the actual level of knowledge, a higher total knowledge score was significantly associated with the completion of COVID-19-related training programs (*B* = 0.181, 95% CI: 0.099 to 0.264, *P* < 0.001), working in a public hospital or clinic (*P* < 0.001), 5 to 10 years of working experience in orthodontics (*P* = 0.006), and having a master's degree (*P* = 0.018). In contrast, male individuals (*P* < 0.001), those who had the experience of treating or caring for COVID-19 patients (*P* < 0.001), as well as those working outside Hubei province (*P* = 0.007) had a significantly lower total knowledge score (Table 4). In addition, the current status of personal orthodontic practice was on the borderline statistical significance, and those who had resumed their work <2 weeks before had a significantly higher knowledge score (*P* = 0.031).

**Table 4:** Results of univariable and multivariable generalized estimating equations (GEE) regression analyses for total knowledge score.

**Variable**	**Univariable analysis**			**Multivariable analysis**		
	***B***	**95% CI**	***P*-value^¶^**	***B***	**95% CI**	***P-*value^¶^**
**Demographics**						
Age			0.977			
Gender						
Female	Reference			Reference		
Male	−0.171	(−0.244, −0.097)	**<0.001**	−0.151	(−0.226, −0.077)	**<0.001**
Profession			0.887			
Years of orthodontic practice			**0.007**			**0.031**
≤5 years	Reference			Reference		
5–10 years	0.146	(0.062, 0.230)	**0.001**	0.124	(0.035, 0.212)	**0.006**
10–20 years	0.063	(−0.022, 0.147)	0.146	0.054	(−0.038, 0.146)	0.247
>20 years	0.013	(−0.096, 0.122)	0.809	−0.012	(−0.131, 0.107)	0.844
Academic degree			**<0.001**			**0.001**
PhD	Reference			Reference		
Master	0.137	(0.010, 0.265)	**0.035**	0.156	(0.027, 0.284)	**0.018**
Bachelor	−0.028	(−0.148, 0.092)	0.645	0.039	(−0.086, 0.164)	0.539
Junior college	−0.207	(−0.368, −0.067)	**0.005**	−0.081	(−0.242, 0.079)	0.321
Technical secondary school	−0.073	(−0.509, 0.364)	0.744	0.156	(−0.362, 0.523)	0.721
Marital status			0.439			
Cohabitants			0.418			
**Workplace information**						
Location			**0.016**			**0.002**
Wuhan	Reference			Reference		
Hubei province (excluding Wuhan)	0.044	(−0.169, 0.257)	0.685	0.065	(−0.140, 0.270)	0.535
Others	−0.133	(−0.249, −0.018)	**0.023**	−0.175	(−0.302, −0.048)	**0.007**
Setting						
Public	Reference			Reference		
Private	−0.231	(−0.303, −0.160)	**<0.001**	−0.174	(−0.253, −0.096)	**<0.001**
Orthodontic services during epidemic			0.199			
Online consultation			0.675			
Current status of orthodontic services			0.417			
**Personal experience**						
Participation in anti-epidemic activities			0.439			
Current status of orthodontic practice			**0.003**			0.050
Not resumed yet	Reference			Reference		
resumed, less than 2 weeks	−0.159	(−0.253, −0.064)	**0.001**	−0.113	(−0.216, −0.010)	**0.031**
resumed, 2 to 4 weeks	0.020	(−0.064, 0.105)	0.640	0.025	(0.124, 0.246)	0.620
resumed, more than 4 weeks	−0.001	(−0.090, 0.089)	0.990	−0.024	(0.081, 0.200)	0.655
No suspension	0.070	(−0.483, 0.623)	0.804	0.109	(0.668, 0.147)	0.701
Completion of COVID−19 related training program						
No	Reference			Reference		
Yes	0.204	(0.125, 0.283)	**<0.001**	0.181	(0.099, 0.264)	**<0.001**
Experience of treating or caring for COVID-19 patients						
No	Reference			Reference		
Yes	−0.265	(−0.429, −0.101)	**0.002**	−0.324	(−0.483, −0.165)	**<0.001**

¶ *P-values in bold are statistically significant (<0.05)*.

### Attitude

In general, most respondents agreed about the effectiveness of PPE. They also admitted that it would be somewhat inconvenient to use PPE while treating patients. Supplementary Table 3 shows the differences in attitude among profession groups. Orthodontic nurses expressed significantly more agreement that PPE could protect orthodontic staff (*P* = 0.010) and orthodontic patients (*P* = 0.001) from COVID-19, and felt less inconvenient to use PPE when treating/caring for patients (*P* < 0.001).

About two-thirds of the participants (64.1%) stated that they were willing to treat or care for patients with confirmed or suspected COVID-19. According to multivariable GEE analysis, the odds of being willing to treat/care for COVID-19 patients were significantly higher among orthodontic nurses (*P* < 0.001), those with a higher self-perceived level of knowledge (*P* < 0.001), those who worked at a public hospital or clinic (*P* < 0.001) that provided online consultation to both existing and potential new orthodontic patients (*P* = 0.001), those with a junior college (*P* = 0.011) or bachelor's degree (*P* = 0.019), and those who had an experience of anti-epidemic activities (*P* = 0.022) (Table 5).

**Table 5:** Results of univariable and multivariable generalized estimating equations (GEE) regression analyses for willingness to treat/care for patients with COVID-19.

**Variable**	**Univariable analysis**			**Multivariable analysis**		
	**OR**	**95% CI**	***P-*value^¶^**	**OR**	**95% CI**	***P-*value^¶^**
**Demographics**						
Age	0.982	(0.973, 0.992)	**<0.001**	1.007	(0.985, 1.030)	0.538
Gender			0.091			
Profession			**<0.001**			**<0.001**
Orthodontist	Reference			Reference		
Orthodontic resident/postgraduate student	1.249	(1.023, 1.526)	**0.029**	1.006	(0.746, 1.357)	0.968
Orthodontic nurse	3.210	(2.429, 4.241)	**<0.001**	1.920	(1.377, 2.678)	**<0.001**
Years of orthodontic practice			**0.001**			0.175
≤5 years	Reference			Reference		
5–10 years	0.836	(0.684, 1.023)	0.082	0.931	(0.725, 1.197)	0.578
10–20 years	0.705	(0.570, 0.870)	**0.001**	0.713	(0.514, 0.989)	**0.043**
>20 years	0.650	(0.495, 0.854)	**0.002**	0.619	(0.372, 1.029)	0.064
Academic degree			**<0.001**			**<0.001**
PhD	Reference			Reference		
Master	0.992	(0.730, 1.349)	0.961	0.967	(0.701, 1.333)	0.837
Bachelor	1.615	(1.203, 2.170)	**0.001**	1.485	(1.069, 2.065)	**0.019**
Junior college	1.801	(1.245, 2.605)	**0.002**	1.781	(1.142, 2.776)	**0.011**
Technical secondary school	1.517	(0.443, 5.193)	0.507	1.952	(0.588, 6.481)	0.274
Marital status			**0.007**			0.273
Single	Reference			Reference		
Married, without children	0.890	(0.660, 1.200)	0.444	0.916	(0.654, 1.283)	0.610
Married, with children	0.754	(0.634, 0.896)	**0.001**	0.824	(0.618, 1.098)	0.186
Others	0.470	(0.194, 1.142)	0.096	0.447	(0.175, 1.141)	0.092
Cohabitants			0.078			
**Workplace information**						
Location			0.623			
Setting						
Public	Reference			Reference		
Private	0.645	(0.541, 0.771)	**<0.001**	0.643	(0.517, 0.799)	**<0.001**
Orthodontic services during epidemic			0.052			
Online consultation			**<0.001**			**<0.001**
No	Reference			Reference		
Provided, only to existing orthodontic patients	1.031	(0.798, 1.332)	0.817	1.091	(0.827, 1.438)	0.538
Provided, to both existing and potential new orthodontic patients	1.658	(1.302, 2.112)	**<0.001**	1.586	(1.219, 2.063)	**0.001**
Current status of orthodontic services			0.847			
**Personal experience**						
Participation in anti-epidemic activities						
No	Reference			Reference		
Yes	1.428	(1.193, 1.710)	**<0.001**	1.270	(1.035, 1.559)	**0.022**
Current status of orthodontic practice			0.929			
Completion of COVID-19 related training program						
No	Reference			Reference		
Yes	1.479	(1.222, 1.790)	**<0.001**	1.176	(0.941, 1.470)	0.153
Experience of treating or caring for COVID-19 patients						
No	Reference			Reference		
Yes	2.046	(1.280, 3.272)	**0.003**	1.348	(0.823, 2.208)	0.235
**Knowledge**						
Self-perceived level of knowledge	1.724	(1.518, 1.957)	**<0.001**	1.705	(1.482, 1.962)	**<0.001**
Total knowledge score	1.026	(0.934, 1.126)	0.595			

¶ *P-values in bold are statistically significant (<0.05)*.

**Figure 3:**
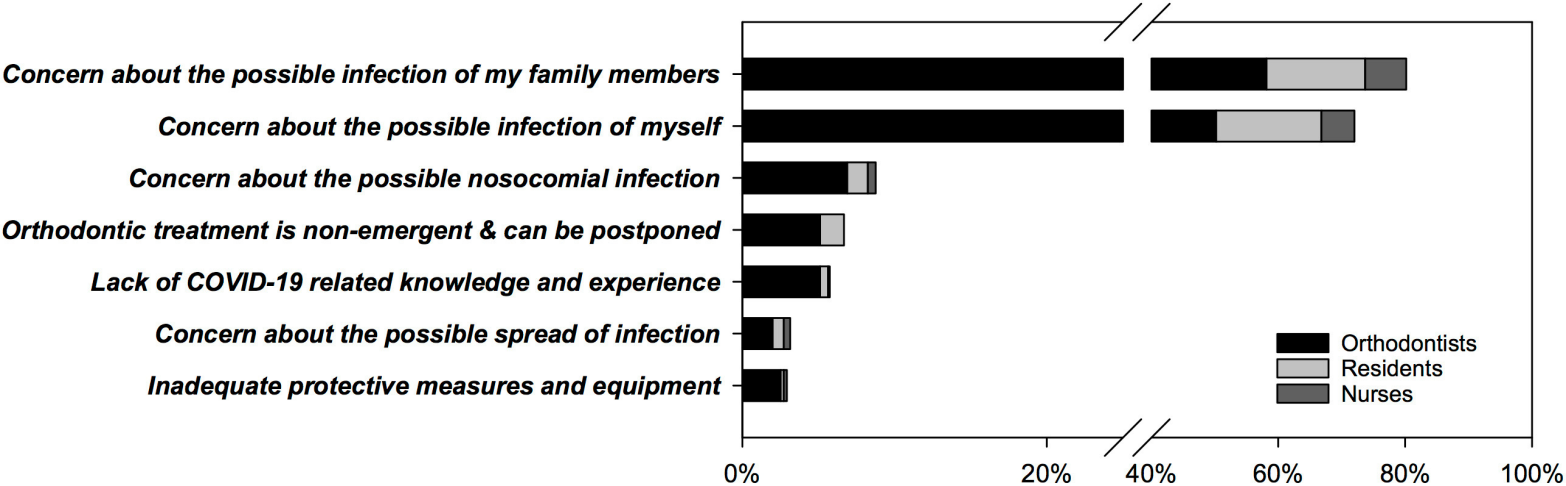
The reasons for being unwilling to treat or care for patients confirmed or suspected with COVID-19 (semi-open question, #31) (*n* = 958).

As depicted in Figure 3, among respondents who were unwilling to treat/care for COVID-19 patients, the most common reasons were concern about the possible infection of their family members (80.2%) and possible infection of themselves (72.0%). Except for the above two choices we provided in this semi-open question, respondents also gave other reasons including concern about possible nosocomial infection (8.8%), the non-emergent nature of orthodontic treatment (6.6%), a lack of COVID-19-related knowledge and experience (5.7%), concern about the possible spread of infection (3.1%), and inadequate protective measures and equipment (2.9%).

### Behaviors

In the final part of the questionnaire, respondents were asked about their behaviors after the resumption of orthodontic services (Supplementary Table 4). Most respondents (72.3%) agreed that their hospitals/clinics had adequate PPE for them to use, and 61.7% reported that they were 100% sure that after work resumption, they would use the recommended PPE during treatment. In addition, orthodontic nurses reported better compliance with PPE (*P* = 0.001) and were less likely to forget to change PPE between patients (*P* < 0.001).

## Discussion

After the announcement of human-to-human transmission and infections among HCWs on January 20, 2020, China imposed more rigorous public health interventions (e.g., compulsory wearing of face masks in public places, intensive intercity and intracity traffic restriction, social distancing, home isolation, and centralized quarantine) to control the spread of SARS-CoV-2 (12). Since around January 27, 2020, relevant government agencies of most provinces and cities in China began to release their regulations on dental practices during the epidemic, which explicitly suspended all elective dental services, including orthodontic treatment (13, 14). About 1 month later, as the number of daily new cases had decreased substantially in China, provinces and cities began to loosen public health interventions and gradually resume non-emergency dental services, according to their own situation. For instance, as the hardest-hit city in China, Wuhan officially lifted its lockdown on April 8, 2020, and its largest dental care provider—Hospital of Stomatology, Wuhan University, started to provide elective treatments on April 20, 2020. Therefore, with 2,669 responses collected from across China during April 11–13, the present study provides valuable insights into the COVID-19-related experiences, knowledge, attitudes, and behaviors of orthodontic professionals during such a period of transition.

The COVID-19 epidemic had a huge impact on orthodontic practice. In this study, a majority of the respondents stated that, during the epidemic, their workplaces had suspended orthodontic treatment (97.8%) and provided online orthodontic consultation to patients (87.4%). These figures are generally in line with those of a previous study conducted during February 17–23, which found that all of the surveyed 48 public tertiary dental hospitals in China had suspended non-emergent dental services, and 33 of them (68.8%) had been offering online dental consultations during suspension (14). Similarly, some other countries implemented mandatory suspension of dental non-emergencies, including orthodontic treatment to fight against the COVID-19 pandemic and ensure the safety of patients and medical staff (23–25).

With the gradual recovery of normal dental services in China, most of the respondents (87.1%) reported that their workplaces had resumed orthodontic services completely or partially. Compared to orthodontists and nurses, significantly fewer orthodontic residents/postgraduate students had resumed orthodontic work, completed any COVID-19-related training programs, or had experience in anti-epidemic activities or treatment for COVID-19 patients, This could be explained by the fact that they are considered students in China, and had been required by the Ministry of Education to remain at home and not to return to their dental schools until further notice (13). In addition, Wuhan and other parts of Hubei had a significantly lower proportion of people who had resumed work. These results are in accordance with the severe situation in Wuhan and other parts of Hubei during the COVID-19 epidemic, as well as the fact that they were the latest in China to loosen public health interventions.

As part of the preparation before work resumption, nearly 80% of the respondents had finished training; three-quarters reported that the training was their primary source of COVID-19-related information. In addition, most respondents also acquired information from the Internet and television during home quarantine. However, only one-third stated that they obtained the relevant knowledge from medical journals. As COVID-19 was an up-to-date topic and most related articles were published in English, language could be a potential barrier to the knowledge acquisition from medical journals for Chinese practitioners. They could turn to other resources, such as authority websites and official notification, for relevant information.

Responses to our questions regarding knowledge have shown a discrepancy between the self-perceived and actual level of knowledge. Although the majority of respondents stated that they understood COVID-19-related knowledge, risks, and protective measures very well, their total knowledge score was generally suboptimal. According to multivariable analyses, after the adjustment of potential confounding variables, the completion of relevant training programs was significantly associated with both more confidence in knowledge mastery and a higher total knowledge score. This is consistent with previous studies, which suggested additional training and education improved dental practitioners' knowledge of infection control (26, 27). This finding highlights the importance for dental hospitals, clinics, and relevant professional societies to organize adequate training activities for HCWs before the resumption of dental services. We also found that the time of work resumption was a borderline predictor of the actual level of knowledge; those who had just resumed work performed better. Although COVID-19-related training was required before work resumption in China, orthodontic professionals may forget relevant knowledge as time elapsed. This possibly suggests that regular training is essential to enhance their knowledge mastery. In addition, although respondents with the experience of treating/caring for COVID-19 patients had higher self-perceived knowledge, they performed significantly poorer in the questions that tested their actual knowledge. The possible reason may be that during the epidemic, the highest level of protection was taken in the treatment. However, in this transition period of work resumption, the focus of dental practice shifted from emergencies to routine procedures, and particular caution and different precautions should be taken.

With regard to the respondents' attitude, 64.1% of them were willing to treat/care for patients with confirmed or suspected COVID-19. Eighty percent of those who were reluctant expressed their concern over possible infection of their family members. Researchers found that fear of transmitting the virus to family and coworkers was the top concern of dentists and frontline HCWs (28). Previous studies have shown that knowledge and education could positively affect dentists' attitudes toward the treatment of patients with highly infectious diseases (27, 29). In the present study, however, both the actual knowledge level and the completion of relevant training programs did not increase respondents' willingness to treat COVID-19 patients. Due to the sudden outbreak and quick spread of COVID-19, as well as there being no vaccine or approved treatment, the fear of the unknown and uncertainty about the disease was the main obstacle to the treatment of COVID-19 patients (30). Home quarantine is crucial to the diffusion of coronavirus, but added difficulties to dental treatment and triggered excessive anxiety (31). We also found that respondents from public dental institutions with online consultation were more willing to treat COVID-19 patients. This is probably because these institutions have adequate prevention measures and sufficient medical sources to protect their staff, while the shortage of PPE is strongly associated with the stress and anxiety of HCWs (32).

In the present study, although many respondents had a suboptimal total knowledge score, most of them believed that they could improve their adherence to PPE. A previous study indicated that dental practitioners were generally ill-prepared for COVID-19, as they did not routinely wear recommended respirators during treatment (33). The use of PPE is crucial for protection, and practitioners need to understand which level of protective measures should be used (34). Hand hygiene is also considered a critical measure for infection control, with poor adherence believed to be a major contributor to disease transmission (35). Researchers found that the implementation of recommended infection control measures varied among dentists, and those who were aware of the importance of infection control had better compliance with guidelines (26). This suggests that the use of effective strategies and different modes of training is necessary to enhance dental professionals' awareness of infection control. It has been recommended that the use of a checklist in handy places for repeatable procedures improves the compliance with infection control precautions, including what PPE should be used in certain circumstances, the correct donning and doffing procedures of PPE, and the recommended hand hygiene measures (36).

A questionnaire-based survey is a useful tool to efficiently acquire information regarding opinions and experiences covering wide demographics of participants (37). In the present study, we obtained a sufficiently large sample for data analysis; however, careful interpretation of the results is required. First, this was an online survey and many participants were recruited via one professor's network, which was a potential source of bias. Second, some categories of the respondents' characteristics had a very small sample size, which caused the imprecision of the results and compromised representativeness of certain populations. In addition, a more thorough investigation into the impact of COVID-19 on orthodontists' provision of healthcare services was out of the scope of this study and therefore not included. This could be explored in future research.

## Conclusions

Results of the present study suggest that, during the mitigation stage of the COVID-19 epidemic when orthodontic services are gradually resuming, orthodontic professionals in China are generally confident that they understand COVID-19-related risks and knowledge, and about two-thirds of them are willing to treat or care for COVID-19 patients. In addition, COVID-19-related training programs are essential for the improvement of knowledge, confidence, and preparedness of orthodontic professionals before work resumption. Sufficient and proper protection should also be provided to ensure safety and reduce the psychological burden on them.

## Data Availability Statement

The raw data supporting the conclusions of this article will be made available by the authors, without undue reservation.

## Ethics Statement

The studies involving human participants were reviewed and approved by Ethics Committee of School & Hospital of Stomatology, Wuhan University. Written informed consent for participation was not required for this study in accordance with the national legislation and the institutional requirements.

## Author Contributions

FH: study conception. FH, DQ, and HH: study design. FH, DQ, JY, TZ, and HH: data collection. FH and DQ: data analysis and manuscript drafting. HH: data interpretation. JY, TZ, and HH: critical revision of the manuscript. All authors: approval of the final version.

## Conflict of Interest

The authors declare that the research was conducted in the absence of any commercial or financial relationships that could be construed as a potential conflict of interest.
