# Supplementary material for: COVID-19 Related Experience, Knowledge, Attitude, and Behaviors Among 2,669 Orthodontists, Orthodontic Residents, and Nurses in China: A Cross-Sectional Survey
Source: Front Med (Lausanne). 2020 Aug 7;7:481. doi: 10.3389/fmed.2020.00481 (PMC7427309; doi:10.3389/fmed.2020.00481)
Supplement: Supplementary file 1 [file Data_Sheet_1.DOCX]

**The Questionnaire Used in This Study**

We are inviting you to participate in a clinical study entitled “COVID-19 related experience, knowledge, attitude and behaviors among orthodontists, orthodontic residents and nurses in China: A survey”. The study protocol has been approved by the Ethics Committee of School & Hospital of Stomatology, Wuhan University (No. 2020-B25).

Before deciding whether to participate in this study, please read the information in the following link as carefully as possible. It can help you understand why we carry out this study, the procedure for it, and related information. You can also discuss with your relatives and friends, if you like, to help you make a decision. **The submission of this questionnaire will be regarded as your consent to participate.**

This survey is anonymous and takes about **5 minutes** to complete.

1. Age: ________
2. Gender
   1. Male
   2. Female
3. Profession
   1. Orthodontist
   2. Orthodontic resident / postgraduate student
   3. Orthodontic nurse
4. Years of orthodontic practice (including postgraduate education period)
   1. ≤ 5 years
   2. 5 to 10 years
   3. 10 to 15 years
   4. > 20 years
5. Highest academic degree
   1. PhD
   2. Master
   3. Bachelor
   4. Junior college
   5. Technical secondary school
6. Location of your workplace: ________
7. Setting of your workplace
   1. Public
   2. Private
8. What was the status of the orthodontic services in your workplace during epidemic?
   1. Complete suspension
   2. Partial suspension
   3. No suspension
9. Did your workplace provide online consultation during epidemic?
   1. No
   2. Provided, only to existing orthodontic patients
   3. Provided, to both existing and potential new orthodontic patients
10. Which anti-epidemic activity did you participate in during epidemic?
    1. None
    2. Support fever clinic
    3. Support designated hospital for COVID-19
    4. Community volunteer
    5. Other (please specify: ________)
11. What is the current status of the orthodontic services in your workplace?
    1. Not resumed yet
    2. Partially resumed
    3. Completely resumed
    4. No suspension
12. What is the current status of the orthodontic services of yourself?
    1. Not resumed yet
    2. Resumed, less than 2 weeks
    3. Resumed, 2 to 4 weeks
    4. Resumed, more than 4 weeks
    5. No suspension
13. Marital status
    1. Single
    2. Married, without children
    3. Married, with children
    4. Others
14. Who are you living with in your workplace? (multiple choices are allowed)
    1. Parents
    2. Children
    3. Spouse
    4. Friends
    5. Fellow students
    6. Colleagues
    7. Other(please specify: ________)
    8. None (live alone)
15. Have you completed the training program about COVID-19?
    1. Yes
    2. No
16. Have you had the experience of treating or caring for patients confirmed or suspected with COVID-19?
    1. Yes
    2. No
17. You understand the relevant knowledge of COVID-19.
    1. Completely agree
    2. Agree
    3. Neither agree nor disagree
    4. Disagree
    5. Completely disagree
18. The sources of your knowledge about COVID-19 include: (multiple choices are allowed)
    1. Television
    2. Newspaper
    3. Internet
    4. Medical journals
    5. Hospital training program
    6. Other (please specify: ________)
19. You are confident that you understand the risks of COVID-19 epidemic for patients and health care workers.
    1. Completely agree
    2. Agree
    3. Neither agree nor disagree
    4. Disagree
    5. Completely disagree
20. You are confident that you understand how to protect yourself and your patients during COVID-19 epidemic.
    1. Completely agree
    2. Agree
    3. Neither agree nor disagree
    4. Disagree
    5. Completely disagree
21. Correct personal protective equipment (PPE) includes: (multiple choices are allowed)
    1. Surgical mask
    2. N95 mask
    3. Isolation gown
    4. Protective clothing
    5. Gloves
    6. Goggles
    7. Face shield
    8. Hand hygiene
22. Hand hygiene includes either washing hands with soap and water, or the use of an alcohol-based hand rub.
    1. True
    2. False
23. It is adequate to use an alcohol-based hand rub if the hands are visibly soiled.
    1. True
    2. False
24. Use of correct PPE eliminates the need for hand hygiene.
    1. True
    2. False
25. When should you wear goggles and a face shield at the same time during treatment? (multiple choices are allowed)
    1. Precheck triage / dental radiology
    2. Oral examination / low risk procedures
    3. Aerosol generating procedures
    4. Suspected or confirmed COVID-19 patients
    5. Waste transportation / apparatus cleaning
26. When could you only wear a surgical mask during treatment? (multiple choices are allowed)
    1. Precheck triage / dental radiology
    2. Oral examination / low risk procedures
    3. Aerosol generating procedures
    4. Suspected or confirmed COVID-19 patients
    5. Waste transportation / apparatus cleaning
27. Use of PPE will keep orthodontic staffs from getting COVID-19.
    1. Completely agree
    2. Agree
    3. Neither agree nor disagree
    4. Disagree
    5. Completely disagree
28. Use of PPE will keep orthodontic patients from getting COVID-19.
    1. Completely agree
    2. Agree
    3. Neither agree nor disagree
    4. Disagree
    5. Completely disagree
29. It is inconvenient to use recommended PPE when treating / caring for patients.
    1. Completely agree
    2. Agree
    3. Neither agree nor disagree
    4. Disagree
    5. Completely disagree
30. Are you willing to treat or care for patients confirmed or suspected with COVID-19 if you have the opportunity?
    1. Yes
    2. No
31. If you choose “no” to the above question, what is the major reason? (multiple choices are allowed)
    1. Concern about the possible infection of yourself
    2. Concern about the possible infection of your family members
    3. Other (please specify: ________)
32. All recommended PPE is readily available in your hospital or clinic.
    1. Completely agree
    2. Agree
    3. Neither agree nor disagree
    4. Disagree
    5. Completely disagree
33. Your head nurse or attending doctor would reprimand you if you did not use PPE when treating or caring for patients.
    1. Completely agree
    2. Agree
    3. Neither agree nor disagree
    4. Disagree
    5. Completely disagree
34. The estimated compliance to recommended PPE during treatment or care of patients after work resumption is:
    1. 0%
    2. 10%
    3. 20%
    4. 30%
    5. 40%
    6. 50%
    7. 60%
    8. 70%
    9. 80%
    10. 90%
    11. 100%
35. You will remove your PPE immediately when you leave the treatment room.
    1. Completely agree
    2. Agree
    3. Neither agree nor disagree
    4. Disagree
    5. Completely disagree
36. You often forget to change PPE between patients.
    1. Completely agree
    2. Agree
    3. Neither agree nor disagree
    4. Disagree
    5. Completely disagree
37. You believe that you can improve the compliance to recommended PPE.
    1. Completely agree
    2. Agree
    3. Neither agree nor disagree
    4. Disagree
    5. Completely disagree
