# Supplementary material for: COVID-19 Related Experience, Knowledge, Attitude, and Behaviors Among 2,669 Orthodontists, Orthodontic Residents, and Nurses in China: A Cross-Sectional Survey
Source: Front Med (Lausanne). 2020 Aug 7;7:481. doi: 10.3389/fmed.2020.00481 (PMC7427309; doi:10.3389/fmed.2020.00481)
Supplement: Supplementary file 2 [file Data_Sheet_2.docx]

**Supplementary Table 1.** Respondents’ personal experience during the COVID-19 epidemic by location

| Personal experience ^§^ | Total (n=2669) | Wuhan (n=267) | Hubei excluding Wuhan (n=98) | Others (n=2304) | *P* value |
| --- | --- | --- | --- | --- | --- |
| Participation in anti-epidemic activities |  |  |  |  | 0.003 |
| No | 1907 (71.4) | 212 (79.4) **^a^** | 62 (63.3) **^b^** | 1633 (70.9) **^b^** |  |
| Yes | 762 (28.6) | 55 (20.6) **^a^** | 36 (36.7) **^b^** | 671 (29.1) **^b^** |  |
| Current Status of orthodontic practice |  |  |  |  | < 0.001 **^¶^** |
| Not resumed yet | 849 (31.8) | 249 (93.3) **^a^** | 51 (52.0) **^b^** | 549 (23.8) **^c^** |  |
| Resumed, less than 2 weeks | 478 (17.9) | 15 (5.6) **^a^** | 22 (22.4) **^b^** | 441 (19.1) **^b^** |  |
| Resumed, 2 to 4 weeks | 725 (27.2) | 2 (0.7) **^a^** | 16 (16.3) **^b^** | 707 (30.7) **^c^** |  |
| Resumed, more than 4 weeks | 611 (22.9) | 1 (0.4) **^a^** | 9 (9.2) **^b^** | 601 (26.1) **^c^** |  |
| No suspension | 6 (0.2) | 0 (0.0) **^a^** | 0 (0.0) **^a^** | 6 (0.3) **^a^** |  |
| Completion of COVID-19 related training program |  |  |  |  | 0.001 |
| No | 553 (20.7) | 78 (29.2) **^a^** | 19 (19.4) **^a, b^** | 456 (19.8) **^b^** |  |
| Yes | 2116 (79.3) | 189 (70.8) **^a^** | 79 (80.6) **^a, b^** | 1848 (80.2) **^b^** |  |
| Experience of treating or caring for COVID-19 patients |  |  |  |  | < 0.001 |
| No | 2564 (96.1) | 243 (91.0) **^a^** | 89 (90.8) **^a^** | 2232 (96.9) **^b^** |  |
| Yes | 105 (3.9) | 24 (9.0) **^a^** | 9 (9.2) **^a^** | 72 (3.1) **^b^** |  |

**§** Displayed as N (%)

**¶** Fisher’ exact test

**a, b, c**: groups with the same letters in the same row are not statistically different (*P* > 0.05) according to *post hoc* tests

**Supplementary Table 2.** Respondents’ COVID-19 related knowledge by profession

| **Questions about knowledge** | **Total (n=2669)** | **Orthodontists (n=1731)** | **Orthodontic residents (n=547)** | **Orthodontic nurses (n=391)** | ***P* value ^¶^** |
| --- | --- | --- | --- | --- | --- |
| Self-perceived level of knowledge **^§^** | 4.03 (0.65) | 4.07 (0.64) **^a^** | 3.80 (0.64) **^b^** | 4.16 (0.65) **^c^** | **< 0.001** |
| 17. You understand the relevant knowledge of COVID-19 | 4 (4 – 4) | 4 (4 – 5) **^a^** | 4 (3 – 4) **^b^** | 4 (4 – 5) **^a^** | **< 0.001** |
| 19. You are confident that you understand the risks of COVID-19 epidemic for patients and HCWs | 4 (4 – 5) | 4 (4 – 5) **^a^** | 4 (4 – 4) **^b^** | 4 (4 – 5) **^a^** | **< 0.001** |
| 20. You are confident that you understand how to protect yourself and your patients during COVID-19 epidemic | 4 (4 – 4) | 4 (4 – 4) **^a^** | 4 (3 – 4) **^b^** | 4 (4 – 5) **^c^** | **< 0.001** |
| Total knowledge score **^§^** | 2.74 (0.85) | 2.75 (0.85) | 2.73 (0.85) | 2.74 (0.86) | 0.887 |
| 21. What is the correct PPE | 51 (1.9) | 30 (1.7) | 13 (2.4) | 8 (2.0) | 0.618 |
| 22. Hand hygiene includes either washing hands with soap and water, or the use of an alcohol-based hand rub | 2347 (87.9) | 1534 (88.6) **^a^** | 491 (89.8) **^a^** | 322 (82.4) **^b^** | **0.001** |
| 23. It is adequate to use an alcohol-based hand rub if the hands are visibly soiled | 1086 (40.7) | 718 (41.5) **^a^** | 183 (33.5) **^b^** | 185 (47.3) **^a^** | **< 0.001** |
| 24. Use of correct PPE eliminates the need for hand hygiene | 2613 (97.9) | 1698 (98.1) | 532 (97.3) | 383 (98.0) | 0.492 |
| 25. When should you wear goggles and a face shield at the same time during treatment | 259 (9.7) | 180 (10.4) | 53 (9.7) | 26 (6.6) | 0.077 |
| 26. When could you only wear a surgical mask during treatment | 958 (35.9) | 593 (34.3) **^a^** | 219 (40.0) **^b^** | 146 (37.3) **^a, b^** | **0.040** |

#17, #19, #20: Displayed as median (25th percentile – 75 percentile). Likert scale, ranging from 1 (completely disagree) to 5 (completely agree)

#21 to #26: Displayed as N (%), number and percentage of correct response

**§** Displayed as mean (SD)

**¶** *P* values in bold are statistically significant (< 0.05)

**a, b, c**: groups with the same letters in the same row are not statistically different (*P* > 0.05) according to *post hoc* tests

**Supplementary Table 3.** Respondents’ attitude towards COVID-19 by profession

| **Questions about attitude** | **Total (n=2669)** | **Orthodontists (n=1731)** | **Orthodontic residents (n=547)** | **Orthodontic nurses (n=391)** | ***P* value ^¶^** |
| --- | --- | --- | --- | --- | --- |
| 27. Use of PPE will keep orthodontic staffs from getting COVID-19 | 4 (3 – 4) | 4 (3 – 4) **^a, b^** | 4 (3 – 4) **^a^** | 4 (3 – 5) **^b^** | **0.010** |
| 28. Use of PPE will keep orthodontic patients from getting COVID-19 | 4 (2 – 4) | 4 (2 – 4) **^a^** | 4 (2 – 4) **^a^** | 4 (3 – 4) **^b^** | **0.001** |
| 29. It is inconvenient to use recommended PPE when treating / caring for patients | 4 (2 – 4) | 4 (3 – 4) **^a^** | 3 (2 – 4) **^b^** | 3 (2 – 4) **^b^** | **< 0.001** |
| 30. Are you willing to treat / care for patients confirmed or suspected with COVID-19 if you have the opportunity | 1711 (64.1) | 1033 (59.7) **^a^** | 355 (64.9) **^a^** | 323 (82.6) **^b^** | **< 0.001** |

#27 to #29: Displayed as median (25th percentile – 75 percentile). Likert scale, ranging from 1 (completely disagree) to 5 (completely agree)

#30: Displayed as N (%), number and percentage of yes response

**¶** *P* values in bold are statistically significant (< 0.05)

**a, b, c**: groups with the same letters in the same row are not statistically different (*P* > 0.05) according to *post hoc* tests.

**Supplementary Table 4.** Respondents’ COVID-19 related behaviors by profession

| **Questions about behaviors ^§^** | **Total (n=2669)** | **Orthodontists (n=1731)** | **Orthodontic residents (n=547)** | **Orthodontic nurses (n=391)** | ***P* value ^¶^** |
| --- | --- | --- | --- | --- | --- |
| 32. All recommended PPE is readily available in your hospital or clinic | 4 (3 – 4) | 4 (3 – 4) **^a^** | 4 (3 – 4) **^a^** | 4 (4 – 5) **^b^** | **< 0.001** |
| 33. Your head nurse or attending doctor would reprimand you if you did not use PPE when treating or caring for patients | 4 (4 – 5) | 4 (4 – 5) | 4 (4 – 5) | 4 (4 – 5) | 0.524 |
| 34. The estimated compliance to recommended PPE during treatment or care of patients after work resumption (%) | 100 (80 – 100) | 100 (80 – 100) **^a^** | 100 (80 – 100) **^a^** | 100 (90 – 100) **^b^** | **0.001** |
| 35. You will remove your PPE immediately when you leave the treatment room | 4 (4 – 5) | 4 (4 – 5) **^a^** | 4 (3 – 4) **^b^** | 4 (4 – 5) **^a^** | **< 0.001** |
| 36. You often forget to change PPE between patients | 2 (2 – 3) | 2 (2 – 3) **^a^** | 2 (2 – 3) **^a^** | 2 (1 – 2) **^b^** | **< 0.001** |
| 37. You believe that you can improve the compliance to recommended PPE | 4 (4 – 5) | 4 (4 – 5) **^a^** | 4 (4 – 4) **^a^** | 4 (4 – 4) **^a^** | **0.013** |

**§** Displayed as median (25th percentile – 75 percentile). Likert scale, ranging from 1 (completely disagree) to 5 (completely agree)

**¶** *P* values in bold are statistically significant (< 0.05)

**a, b, c**: groups with the same letters in the same row are not statistically different (*P* > 0.05) according to *post hoc* tests.
